# Facile Synthesis of Ni-Doped ZnO Nanoparticles Using Cashew Gum: Investigation of the Structural, Optical, and Photocatalytic Properties

**DOI:** 10.3390/molecules28237772

**Published:** 2023-11-25

**Authors:** Alexsandro Lins, Aimee G. Jerônimo, Ricardo Barbosa, Luan Neves, Pollyana Trigueiro, Luciano C. Almeida, Josy A. Osajima, Francisca A. Pereira, Ramón R. Peña-Garcia

**Affiliations:** 1 Unidade Acadêmica do Cabo de Santo Agostinho, Programa de Pós-Graduação em Engenharia Física, Universidade Federal Rural de Pernambuco, Cabo de Santo Agostinho 52171-900, PE, Brazil; alexlins1500@gmail.com (A.L.); aimeejeronimo@gmail.com (A.G.J.); ricardojunior1902@gmail.com (R.B.); luan.neves@ufrpe.br (L.N.); pollyanatrigueiro@gmail.com (P.T.); araujofp15@gmail.com (F.A.P.); 2 Departamento de Engenharia Química, Universidade Federal de Pernambuco, Recife 50670-901, PE, Brazil; luciano.calmeida@ufpe.br; 3 Universidade Federal de Piauí, Teresina 64049-550, PI, Brazil; josyosajima@ufpi.edu.br

**Keywords:** green synthesis, Ni-doped ZnO, natural polysaccharides, cashew gum, photocatalysis

## Abstract

This work adopted a green synthesis route using cashew tree gum as a mediating agent to obtain Ni-doped ZnO nanoparticles through the sol–gel method. Structural analysis confirmed the formation of the hexagonal wurtzite phase and distortions in the crystal lattice due to the inclusion of Ni cations, which increased the average crystallite size from 61.9 nm to 81.6 nm. These distortions resulted in the growth of point defects in the structure, which influenced the samples’ optical properties, causing slight reductions in the band gaps and significant increases in the Urbach energy. The fitting of the photoluminescence spectra confirmed an increase in the concentration of zinc vacancy defects (V_Zn_) and monovacancies (Vo) as Zn cations were replaced by Ni cations in the ZnO structure. The percentage of V_Zn_ defects for the pure compound was 11%, increasing to 40% and 47% for the samples doped with 1% and 3% of Ni cations, respectively. In contrast, the highest percentage of V_O_ defects is recorded for the material with the lowest Ni ions concentration, comprising about 60%. The influence of dopant concentration was also reflected in the photocatalytic performance. Among the samples tested, the Zn_0.99_Ni_0.01_O compound presented the best result in MB degradation, reaching an efficiency of 98.4%. Thus, the recovered material underwent reuse tests, revealing an efficiency of 98.2% in dye degradation, confirming the stability of the photocatalyst. Furthermore, the use of different inhibitors indicated that •OH radicals are the main ones involved in removing the pollutant. This work is valuable because it presents an ecological synthesis using cashew gum, a natural polysaccharide that has been little explored in the literature.

## 1. Introduction

In recent decades, the development of industrial sectors focused on textile, cosmetic, and graphic manufacturing has played a crucial role in the economies of several countries [1,2]. These sectors are crucial to meet consumer demand, but they can also pose significant risks to the environment, particularly water resources. This can lead to adverse impacts on ecosystems and the health of the population that depends on them [3,4]. Much of this is due to the widespread use of organic dyes, most of which are toxic, combined with the improper disposal of effluents without any treatment, generating the contamination of large volumes of water in rivers and groundwater [5,6,7]. Methylene blue (MB), a phenothiazine derivative commonly used by the textile industry for dyeing fabrics, stands out among the most popular dyes [8,9]. In addition to inhibiting the presence of dissolved oxygen in aquatic environments and harming the fauna present [10], this compound also manifests itself as a potential carcinogen linked to other negative implications for human health, as discussed in Ramsay et al. [11]. The issue of contamination is particularly complicated because of the challenges involved in eliminating pollutants. One such pollutant, which is cationic and contains a heterocyclic aromatic chain that is difficult to break down, is resistant to natural degradation and persists in the environment [12].

There are many ways to remove dyes and other organic pollutants from wastewater, but advanced oxidative processes (AOPs) are highly effective. These methods show great promise in solving pollution problems by breaking down contaminant molecules [13]. Part of the AOPs is heterogeneous photocatalysis, the implementation of which is based on the use of semiconductors, which are photoactivated by UV and visible radiation through the excitation of electrons from the valence band to the conduction band [14,15]. This process generates various oxygenated radicals, including the hydroxyl radical (•OH), which promotes the degradation of contaminating molecules, resulting in non-toxic products and, in many cases, complete mineralization [16,17].

Among the various semiconductors used in photocatalysis, zinc oxide (ZnO) has gained prominence. The synthesis of this semiconductor can be carried out by different methods, such as physical [18], hydrothermal [19], solvothermal [20], sol–gel [21], precipitation [22], and many others. In general, ZnO synthesis is carried out under controlled conditions to ensure the purity of the compound and obtain the desired properties [23]. Furthermore, ZnO synthesis can be adjusted to obtain different particle shapes and sizes, which can influence, for example, its optical and surface properties [20,24,25,26]. In addition, the growth of ZnO oxides using plant derivatives has emerged as a green synthetic approach in recent years [27,28]. Vegetable gums are polysaccharides that act as stabilizing agents for nanoparticles in general, impacting the morphology and size of this nanostructure [29,30]. Studies have reported the use of Acacia [31], Karaya and Arabic [32], Tragacanth [33], and Cashew [34] gums in the synthesis of ZnO. Furthermore, ZnO nanostructures obtained using vegetable gums showed satisfactory photocatalytic results. Araújo et al. [35] used the sol–gel method to manufacture ZnO with a nano-flower-like structure from Karaya gum and reported effective performance in the degradation of methylene blue (MB) dye.

On the other hand, the ZnO properties can be adjusted by dopant inclusion to meet specific requirements for various applications. When it comes to photocatalytic activity, doping the ZnO lattice is crucial for trapping charge carriers from holes and photogenerated electrons in the valence and conduction bands, respectively. This makes recombination difficult, thus ensuring efficient performance [36,37,38]. This allows the species to remain available for longer redox reactions at the surface of the material. Different transition metals have been explored as dopants of the ZnO lattice, and the effect of each of them on the general properties depends on factors such as the synthesis method and the cation concentration in the lattice [39,40,41,42]. In this sense, the use of nickel as a doping element has attracted the attention of numerous researchers due to the improvement caused by the photocatalytic activity of several dyes. Zyoud et al. [43] show that Ni-doped ZnO increased MB degradation under blue light compared to undoped compounds. The inclusion of Ni in the ZnO structure also improved the removal of rhodamine B (RB), as reported by Zhao et al. [44].

The main objective of this study is to synthesize the pure and doped ZnO using different concentrations of Ni cations (1% and 3%) in the presence of natural polysaccharide (cashew gum) as a green mediating agent. Particularly, it was synthesized by the sol–gel route in the presence of cashew gum the Zn_1−x_Ni_x_O compound (x = 0.00, 0.01, and 0.03) and investigated the effects of dopant on the structural, optical, morphological, and photocatalytic properties of ZnO. The photocatalytic activity was evaluated using methylene blue (MB) as a model contaminant under a UV source. Additionally, this study investigated the stability and reaction mechanisms of the Ni-doped ZnO samples. The dopant concentrations and synthesis method were chosen to strictly control synthesis parameters and avoid exceeding solubility limits, while also being cost-effective.

## 2. Results and Discussion

### 2.1. Structural Analysis of the Zn_1−x_Ni_x_O Compound

**Figure 1:**
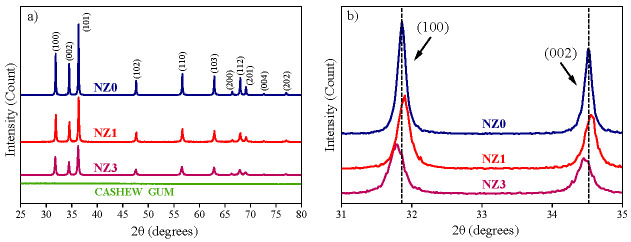
(**a**) XRD patterns for undoped and Ni-doped ZnO samples. (**b**) Magnification between 31° and 35°, for the crystallographic (100) and (002) planes.

The investigation of the structural parameters and phase purity of undoped and Ni-doped ZnO nanoparticles was carried out using the XRD technique (Figure 1). The diffraction patterns for the NZ0, NZ1, and NZ3 samples, as well as for cashew tree gum (CG), are shown in Figure 1a. Firstly, it is noted that there are no diffraction peaks along the spectrum belonging to CG, which confirms the absence of impurities in the polysaccharide and makes it suitable for the synthesis process. On the other hand, in all XRD patterns of the ZnO samples, the diffraction peaks corresponding to the reflection of (100), (002), (101), (102), (110), (103), (200), (112), (201), (004) and (202) planes appear, which are indexed to the wurtzite hexagonal structure of ZnO according to JCPDS data (Card No. 36-1451). Throughout the XRD patterns, reductions in the intensity of the diffraction peaks are noted as the Ni concentration increases by up to 3% in the ZnO structure, a result analogous to that presented by Anbuselvan et al. [45]. These reductions are highlighted in Figure 1b, where the (100) and (002) planes are shown. The addition of dopant ions resulted in a reduction in sample crystallinity, which is reflected in the observed intensity variation. Still, as shown in Figure 1b, slight variations in the 2θ positions for the (100) and (002) planes can be noted, suggesting the Ni^2+^ inclusion into the ZnO crystal structure [46] and their influence on the atomic distortions.

In addition, the diffraction patterns of pure and doped NPs confirm the absence of impurities. This indicates the preservation of the ZnO single-phase wurtzite structure, even after doping. This infers that doping occurs in a substitutional manner, with Ni ions occupying the crystallographic sites of Zn^2+^ [47]. These data are plausible considering the proximity of the ionic radii of Ni (0.069 mm) and Zn (0.074 nm) [48]. On the other hand, it is worth noting that there is a chance that the dopant ions may occupy interstitial positions within the crystal lattice, particularly for doping levels of 3%. This is supported by the slight increase observed in the lattice parameters *a* and *c*, as demonstrated in Table 1. The lattice parameters were estimated from diffraction patterns and using the Equation (1):

(1) $${\left(\frac{1}{d_{hkl}}\right)}^{2}=\frac{4}{3}\left(\frac{h^{2}+hk+k^{2}}{a^{2}}\right)+\frac{l^{2}}{c^{2}},$$

where *a* and *c* are the desired lattice parameters; *h*, *k*, and *l* are the Miller indices, and *d_hkl_* is the spacing distance between the planes (*hkl*) obtained by the formula 2*d_hkl_*sinθ = nλ, with λ being the wavelength of CuKα radiation (1.5406 Ǻ) [49]. To facilitate the calculations, the diffraction peaks corresponding to the (100) and (002) planes were used to find *a* and *c*, respectively [48]. Also, from the *a*/*c* ratio, we again confirmed the maintenance of the hexagonal structure since the values are practically constant for all samples (Table 1) [50]. 

On the other hand, the average crystallite size (*D*) was calculated using the Willianson–Hall method via Equation (2) [51].

(2) $$\beta_{hkl}\cos\left(\theta \right)=\frac{K\lambda }{D}+4\varepsilon \sin\left(\theta \right)$$

Here, *K* is the shape factor whose value is 0.9; *ε* is the lattice microstrain; *β_hkl_* is the total width of half the maximum (FWHM), and θ is the diffraction angle in radians. From the calculated values (Table 1), we noticed a slight reduction in the crystallite for the sample doped with 1% of Ni compared to undoped ZnO. This result agrees with data recorded in other texts [49,52] due to the smaller ionic radius of Ni, which replaces Zn cations, causing slight defects in the host lattice and favoring the crystallite size reduction. However, when the dopant concentration is adjusted to 3%, a significant increase in *D* is observed compared to previous values. Al-Ariki et al. [53] also present an analogous result for the same percentage of Ni concentration. A possible explanation for this involves the occupation of interstitial positions by dopant cations [48], a reason that is also attributed to the increase in unit cell volume and lattice parameters, as discussed previously. Linked to this are lattice tensions and stress, which, according to Peña-Garcia et al. [39], may also be related to crystallite growth. Using the values of a and c and the potential parameter of the hexagonal structure *u* = *a*^2^/3*c*^2^ + 0.25, the bond length (*L*) was determined via Equation (3) [39].

(3) $$L=\sqrt{\left(\frac{a^{2}}{3}+{\left(\frac{1}{2}-u\right)}^{2}c^{2}\right)}$$

In this case, slight variations in *L* values are observed as the dopant increases and may be a consequence of changes in the lattice connection symmetry resulting from defects created by the dopant cations insertion. Following the same trend observed for variations in *D* values, here, there is a slight reduction in *L* from 1.9725 Å (NZ0) to 1.9706 Å (NZ1), slightly increasing to 1.9764 Å (NZ3). These variations may be due to the difference in ionic radii between Zn and Ni cations, as well as the arrangement of Ni cations in the hexagonal structure.

The dislocation density (*δ*) of the Zn_1−x_Ni_x_O compound was estimated by the Equation (4) [54]:

(4) $$\delta =\frac{1}{D^{2}}$$

Based on the values presented in Table 1, we observed a contrary trend to what was observed for the crystallite size (*D*) and bond length (*L*). Specifically, the value of *δ* exhibited a minor increase for 1% doping but decreased significantly with the addition of 3% Ni, as compared to pure ZnO. It is known that the dislocation density is associated with the degree of defects or crystallographic irregularities present in the sample [55]. By this logic, the sample doped with 3% may present a smaller number of surface defects compared to the other compounds, an effect similarly presented in Sankar et al. [56]. On the other hand, increases are recorded in the values of the parameter referring to the network voltage (ε), suggesting that the inclusion of cation causes deformations in the host structure [57], favoring the emergence of deeper defects already mentioned.

### 2.2. Raman Analysis of the Zn_1−x_Ni_x_O Compound

**Figure 2:**
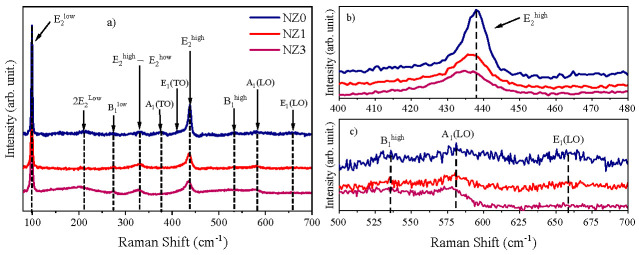
(**a**) Raman spectra for the Zn_1−x_Ni_x_O system; (**b**) broadening of the E_2_^high^ peak, and (**c**) broadening of the B_1_^high^, A_1_(LO) e E_1_(LO) modes.

As mentioned earlier, the inclusion of Ni cation in ZnO can induce defects and structural disturbances in the host lattice and alter the crystalline quality of the compounds synthesized. To understand the nature of the modifications in the material, it is highly recommended to use the Raman spectroscopy technique. Figure 2a displays the Raman spectra of undoped and Ni-doped ZnO in a range of 80–700 cm^−1^. Furthermore, the peaks related to the vibrational modes of the wurtzite structure are marked. It is known from the literature [58] that wurtzite ZnO belongs to the P63mc space group, having optical phonons in the Brillouin zone whose irreducible representation is of the form Г_Opt_ = A_1_ + 2B_1_ + E_1_ + 2E_2_, where A_1_ and E_1_ are the active polar modes in Raman and infrared. Both of these can be divided into transverse (TO) and longitudinal (LO) optical phonons; E_2_ corresponds to nonpolar modes; only Raman is active, and B_1_ modes are considered Raman inactive [59].

For the Zn_1−x_Ni_x_O system, the occurrence of two more intense peaks stands out, which are attributed to the low and high-frequency E^2^ modes, E_2_^low^ and E_2_^high^, at ~99 cm^−1^ and ~435 cm^−1^, respectively. The E_2_^low^ mode occurs due to the vibrations of the zinc sublattice, while the vibration of the oxygen atom is mainly associated with the E_2_^high^ mode [47,60]. In a similar way to that described by Fifere et al. [50], this last phonon is linked to the high crystallinity of the material. In addition, the reduction in its intensity along with its broadening, as observed throughout Figure 2b, infers a reduction in the crystallinity of the compounds through the inclusion of Ni ions in the ZnO crystal lattice, data ratified by the information provided in the XRD analyses. From Figure 2b, it is possible to attest to the Zn by Ni cations replacement in the crystal lattice since the E_2_^high^ mode undergoes slight shifts toward lower wave numbers as the Ni content increases [46]. This tendency to shift to lower wavenumbers is also seen for other peaks, as in the case of the mode attributed to the E_2_^high^ − E_2_^low^ difference, being centered at ~331 cm^−1^ for NZ0 and at ~329 cm^−1^ and ~327 cm^−1^ for NZ1 and NZ3, respectively [61], as well as for phonons 2E_2_^low^ and B_1_^low^, whose values are also indicated in Table 2.

One hypothesis for these changes may be linked to the disorder in the structure of the materials, resulting from the growth of oxygen vacancies (V_O_) in the crystalline lattice due to dopant inclusion, leading to modifications in the bonding symmetry of the atoms, which is also reflected in the slight changes in chemical bond lengths (*L*), as shown in Table 1 [59,62]. The insertion of Ni cations may lead to the growth of interstitial defects and vacancies, which could be the reason behind the disappearance of the A_1_(TO) and E^1^(TO) modes. These modes are only observed in the pure ZnO spectrum at around 377 cm^−1^ and 411 cm^−1^, respectively. Once again, defects can attenuate crystal lattice symmetry, rendering some vibrational modes inactive as they are linked to higher symmetry structures [63]. In Figure 2c, the magnified spectra between 500–700 cm^−1^ display three low-intensity peaks that are now more clearly identified as B_1_^high^, A_1_(LO), and E_1_(LO) modes [63,64]. The intensity of the first and last phonons is lowered due to the dopant ions, possibly due to the vibration limitations of the lattice, which may occur because of the emergence of vacancy defects. On the other hand, vacancy defects may promote an increase in the vibration amplitude of the A_1_(LO) mode.

### 2.3. Investigation of the Optical Properties of the Zn_1−x_Ni_x_O System

**Figure 3:**
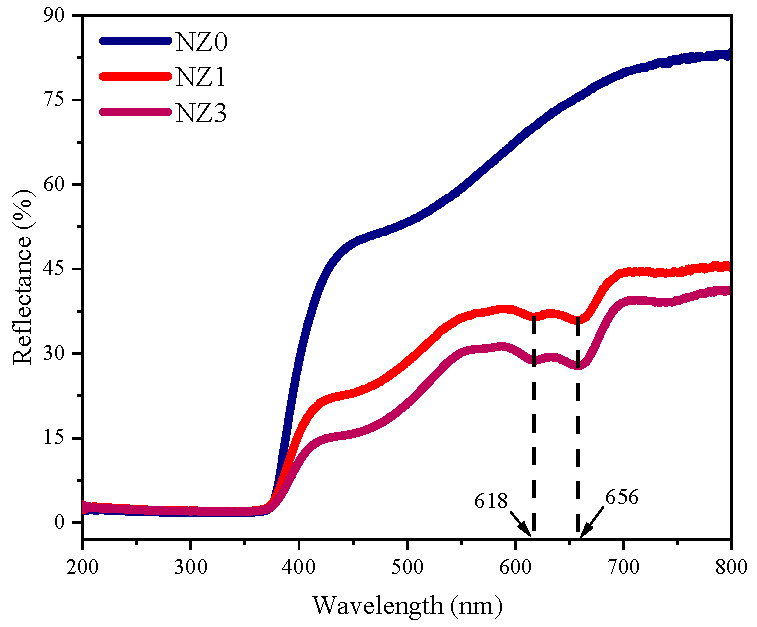
DRS spectra for undoped and doped ZnO samples with 1% and 3% of Ni cations.

The study of the optical properties of the Zn_1−x_Ni_x_O system was carried out using the diffuse reflectance spectroscopy (DRS) technique, whose spectra were obtained in a scanning zone of 200–800 nm, as indicated in Figure 3. It is evident that in the visible region, the reflectance curves of samples NZ1 and NZ3 exhibit absorption bands centered at ~618 nm and ~656 nm, which are not present in NZ0. According to Singh et al. [65], this is related to the internal electronic *d–d* transitions of Ni ions in tetrahedral symmetries, corresponding to the ^3^*T*_1_(*F*) *→* ^3^*T*_1_(*P*) ligand field transitions. There is an increase in reflectance intensity for longer wavelengths, which is also noticeable throughout the entire spectrum of pure ZnO at wavelengths greater than 400 nm. This trend has been similarly reported by Handan et al. [66]. The authors attribute this effect to the increase in the interactions of incident photons with the electronic structures of the synthesized compounds.

Another important aspect to be observed concerns the decrease in reflectance intensity as the doping percentage increases, a fact that confirms the Ni atoms insertion in the wurtzite structure [45] and which can be attributed to the lattice strain, as well as the intermediate energy levels formation in the semiconductor band [67,68]. This last factor is greatly corroborated by the variation in band gap energy (*E_g_*) values of doped ZnO systems compared to undoped ones. To estimate the *E_g_* values, the Tauc model was employed through Equation (5) [69]:

(5) $$F\left(R\right)h\nu ={A\left(h\nu -E_{g}\right)}^{n}$$

where *A* is an energy-independent constant; *hν* is the energy of the incident photon; *n* = 2 is an index referring to the type of direct optical transition, and *F*(*R*) corresponds to the Kubelka–Munk (K–M) function that is expressed through the following Equation (6) [70]:

(6) $$F\left(R\right)=\frac{{\left(1-R\right)}^{2}}{2R}=\frac{K}{S}$$

with *K* and *S* being, respectively, the K–M mirroring and absorption coefficients.

**Figure 4:**
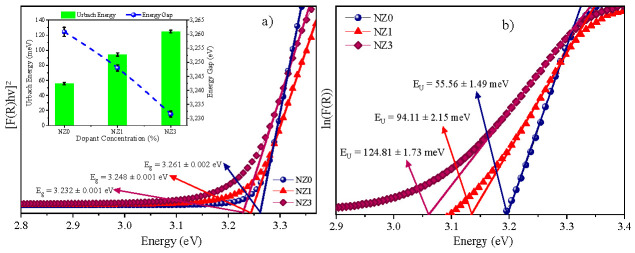
(**a**) Energy band gap and (**b**) Urbach energy. In (**a**), a second graph is inserted in which, on the left scale, are the Urbach energy values (meV) and on the right, the energy gap values (eV), all as a function of the incident energy, *hν*.

Therefore, the energy gap values (*E_g_*) were obtained by extrapolating the linear portion of the square graph of the absorbed energy, [*F(R)hν*]^2^, as a function of the energy of the incident photon, *hν*, as shown in Figure 4a. The impact of Ni dopant on the energy bandgap (*E_g_*) can be observed in this case. The values of *E_g_* for the pure compounds and those doped with 1% and 3% Ni were 3.26 eV, 3.25 eV, and 3.23 eV, respectively. Although the reduction in *E_g_* is small, it can be attributed to various factors. A possible explanation for the reduction in *E_g_* values is the creation of unoccupied states in the gap between the valence and conduction bands of the semiconductor. These states act as electronic traps, capturing excited electrons. These states can be induced by the segregation of the grain boundaries of Ni cations [71], as well as by electron exchange interactions that occupy the *sp–d* orbitals of Ni ions [39,46]. In addition, during synthesis, oxygen and zinc vacancy defects increase, which may be related to the decrease in *E_g_* due to the different characteristics of the cations involved in the system [71,72].

As the doping increases, the energy gap decreases. However, there is a considerable increase in the Urbach energy values. For instance, the Urbach energy value is 55.55 meV for pure ZnO. But for ZnO doped with 1% and 3% Ni, it increases to 94.11 meV and 124.81 meV, respectively. This information is shown in Figure 4a. This trend of increasing E_U_ is entirely plausible, considering that the Urbach energy measures the general disorder present in the material caused by the insertion of doping impurities into the host microstructure [68]. Increasing the percentage of Ni in ZnO leads to a greater imbalance of charges in the samples. This, in turn, promotes an increase in the oxygen (V_O_) and zinc (V_Zn_) vacancies, which, in turn, represents an increase in the disorder in the crystalline lattice [73]. In this way, these disorders can modify the bonding scheme between the atoms and/or ions of the semiconductor and can, consequently, generate states located in the forbidden energy region, which are also known as Urbach tail states. Therefore, the Urbach energy corresponds to the width of these tail states [66,68] and, in this work, it was stipulated from the inverse slope of the linear adjustment line of the curves *ln*(*F*(*R*)) versus the energy *hν*, according to Figure 4b.

**Figure 5:**
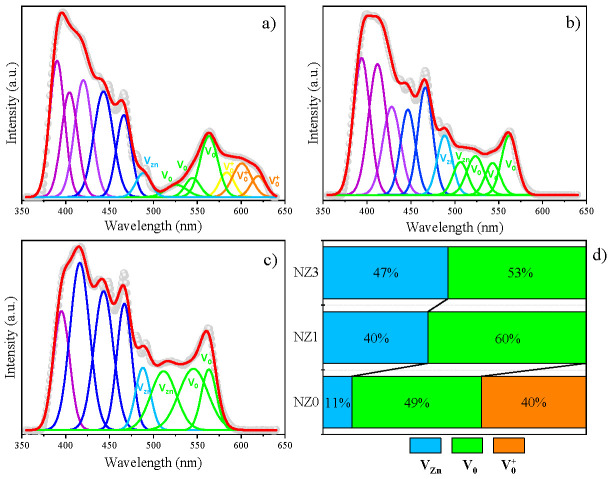
Deconvolution of PL spectra for the Zn_1-x_Ni_x_O system (**a**) NZ0, (**b**) NZ1, (**c**) NZ3. In (**d**), the relative percentages of defects present in each sample are displayed from the bar graphs.

To examine specific defects in the Ni-doped ZnO crystal structure, we performed photoluminescence (PL) measurements at room temperature. The emission spectra obtained are shown in Figure 5. These defects were distinguished and quantified through the deconvolution of the PL curves through a Gaussian distribution function. In principle, a common factor in the spectra of the three samples is the occurrence of a broad and intense band of UV emission followed by a less intense band of visible emission. However, the position and intensity of its peaks end up changing as the percentage of dopants increases. This can be seen, firstly, along the UV band where there is a clear shift of the highest intensity peak to longer wavelengths, being centered at ~390 nm for NZ0, ~394 nm for NZ1, and ~416 nm for NZ3. Furthermore, a clear increase in blue emission related to peaks located in the range of 443–466 nm is also seen upon increasing doping. All these data suggest an increase in the surface defect states for the doped samples since the peaks located in this range result from the near band-edge transition (NBE) as a consequence of the excitonic recombination of electrons located in states close to the edge of the layers conduction and valence [12,46,74,75].

Throughout the visible region, there has been a noticeable increase in photoluminescence (PL) emission. This trend is particularly prominent in the range from 470 nm to 630 nm and is attributed to intrinsic defects such as zinc (V_Zn_) and oxygen (V_O_) vacancies in the ZnO wurtzite network. This increase in emission is caused by the transfer of charges in the forbidden energy range from energy levels located further away from the conduction and valence bands [25,75]. According to the literature [61,76], V_Zn_ vacancies are electron acceptors, while V_O_ is the donor type. Given the above, the percentage quantification of emerging defects in each sample is displayed along the bar graph in Figure 5d. The presence of V_Zn_ defects increases noticeably with doping and accounts for about 11% of the total vacancies found in pure ZnO. In NZ1 and NZ3, the research shows percentages of V_Zn_ defects of 40% and 47%, respectively. This increase in V_Zn_ defects is directly reflected in the cyan emission peak at ~488 nm, which is higher in the doped compounds compared to the undoped ones. Moreover, the appearance of green emission peaks in NZ1 and NZ3 centered at ~506 nm and ~511 nm, respectively, are also attributed to V_Zn_ defects, as shown in Figure 5b,c. The increase in green light emission, particularly in the range of 520 nm to 565 nm, is linked to the presence of neutral oxygen vacancies (V_O_) in the compounds. These vacancies are dominant in all compounds, and their percentage variations are displayed in Figure 5d. The graph shows an increase in V_O_ for ZnO doped with 1% of Ni, which decreases in 3% of Ni. Patwari et al. [77] have reported that the increase in PL curves in the range of vacancy defects may be due to the multiplication of *•OH* groups linked to the surface of compounds. This factor contributes significantly to the semiconductor’s photocatalytic activity.

The photoluminescence (PL) spectrum of pure ZnO shows the presence of single-charged oxygen vacancies, known as V_O_^+^. These vacancies make up about 40% of the total vacancies in the structure and are responsible for producing yellow emissions at around 583 nm and orange–red at around 600 nm and 619 nm (as shown in Figure 5a). In contrast, compounds modified with Ni lack this type of vacancy. The growth of V_Zn_ and V_O_ vacancies in the NZ1 and NZ3 samples extinguishes this type of defect. One explanation for the disappearance of defects in doped compounds can be correlated to the differences in the electronegativity and ionic radii of Ni ions compared to Zn ions. This creates an imbalance of charge in the host microstructure, which changes the bonding scheme between the atoms forming the wurtzite structure. As a result, defects that were present in the undoped structure may be attenuated due to the emergence of new disorders. This can lead to the expansion of already existing defects.

### 2.4. Morphological Analysis of the Zn_1−x_Ni_x_O system

**Figure 6:**
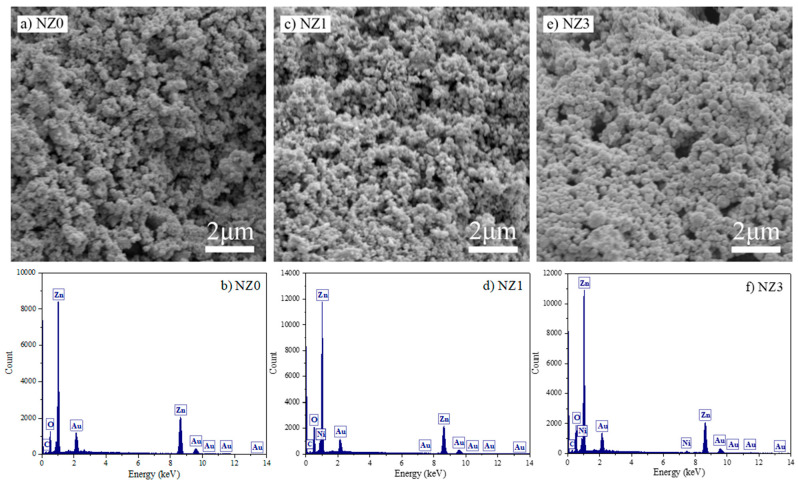
SEM images and EDS spectra for the NZ0, NZ1, and NZ3 samples synthesized by the sol–gel method.

The SEM micrographs of the Zn_1−x_Ni_x_O system synthesized by the sol–gel method are shown in Figure 6. The morphology of the three samples indicates the structuring of homogeneous clusters formed by spherical ZnO nanoparticles. Similar structures were presented by Robles-Águila et al. [78] for ZnO doped with Al and Ni and by Rahmati, A. et al. [79] for Cu-doped ZnO. It is believed that factors such as synthesis route, pH value, and calcination temperature have a major influence on the final morphology of the compounds [12,26,80]. In addition, it is important to highlight that the use of cashew gum may have acted as an inhibitor or mediating agent for the growth of particles. However, considering that all samples were obtained under these same synthesis conditions, it is estimated that the surface morphology is unchanged [81]. Therefore, even though no significant changes were found in the morphology of the pure compounds and those doped with 1% Ni, as shown in Figure 6a,c, it can be seen in Figure 6e that the doping at 3% could cause an increase in the particle size of the semiconductor, which is consistent with the crystallite size results calculated from the XRD patterns.

Alongside the SEM images, the EDS spectra are noteworthy as they enable mapping and analysis of the constituent elements in each sample. The spectra confirm the formation of the hexagonal wurtzite structure that is characteristic of ZnO, based on the presence of dominant peaks corresponding to zinc and oxygen (Figure 6b,d,f). Additionally, the cationic doping in the crystal lattice is confirmed through the presence of peaks that are typical of Ni (Figure 6d,f). The XRD analyses are further supported by this data. A series of peaks are present, primarily related to the elements Au, which originate from the sample coating, and C, which is typical of the sample holder.

### 2.5. Photocatalytic Tests of the Zn_1−x_Ni_x_O System

**Figure 7:**
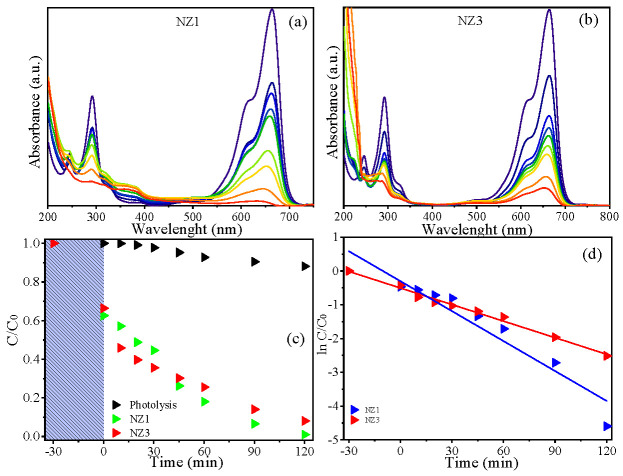
Spectral variation in photocatalysis test using (**a**) NZ1 and (**b**) NZ3 samples, (**c**) C/C_0_ ratio, and (**d**) kinetic of MB degradation.

To evaluate the photocatalysis effectiveness of the NZ1 and NZ3 samples, MB dye was used as a model pollutant. This study was conducted by observing the changes in the 664 nm absorbance band of the MB dye. The results were then plotted, indicating the relationship between concentration rate and irradiation time. Figure 7 displays the plotted results. It was observed that exposing the materials to light caused a reduction in the intensity of the 664 nm band of the MB dye during the experiments (Figure 7a,b). This effect was more pronounced for the NZ1 sample. After 120 min of UV irradiation, the photodegradation efficiency (%) of the dyes was calculated. The MB degradation/discoloration rate was 11.9% in photolysis. However, the calculated degradation/discoloration rate in photocatalysis using the NZ1 and NZ3 samples was 98.4% and 87.8%, respectively. The *C/C*_0_ ratio (Figure 7c) related to the photocatalytic tests performed showed that the curve decreased to close to zero on the *y*-axis, indicating the effectiveness of the material in removing the target dye, especially for the NZ1 sample. The photodegradation kinetics of MB by ZN1 and ZN3 were plotted as shown in Figure 7d. 

The rate constant was determined by finding the slope of the line obtained by plotting *ln*(*C*/*C*_0_) versus irradiation time. The reaction demonstrated a typical profile at pseudo-first order with *k* values of 2.95 × 10^−2^ and 1.63 × 10^−2^ min^−1^ for the NZ1 and NZ3 samples, respectively. These results confirm that the material doped with 1% nickel has a higher reaction rate and, therefore, a greater potential for dye removal in solution. By way of comparison, in Table 3, the values of the reaction rate constant, *k*, obtained for NZ1, NZ3, and other ZnO samples discussed in some texts in the literature for MB removal are shown. The optimal concentration of nickel dopant may have played a role in achieving these photocatalytic results. The PL and Urbach energy results showed that the insertion of nickel promoted the formation of structural defects, mainly oxygen vacancies, and the formation of new energy levels that become electron trappers, reducing recombination and favoring photocatalytic activity.

Dopant cations in the ZnO lattice can cause the formation of the impurity level. A consequence of this is that the photogenerated charge carriers remain available longer to improve the photocatalysis performed [85,86]. From the results of photocatalysis in this study, it was observed that there was an optimized concentration of Ni dopant for ZnO to present a high photocatalytic efficiency. These results are consistent with other studies previously reported in the literature [47,87,88]. For example, Pr-doped ZnO, synthesized by sol–gel, was investigated by Ahmad et al. [89] for photodegradation of methyl orange under visible light radiation. In this study, the material obtained an excellent response in visible light for degradation of almost 90% of the dye after 90 min. Modi et al. [90] developed W-ZnO and Sb-ZnO through the direct precipitation method and applied them to the photodegradation of methylene blue under UV and solar light irradiation. The authors observed a discoloration of up to 91% of the dye solution during 120 min of sun exposure. In studies reported by Bouarroudj et al. [91], Ce and Ag co-doped ZnO, obtained by the hydrothermal method, were used for the degradation of metronidazole under solar light radiation. The authors observed that ZnO co-doped achieved up to 97 and 99% degradation of paracetamol and metronidazole, respectively, in 180 min of reaction. In addition to ZnO, other materials have also stood out as efficient photocatalysts. An example of this is reported by Wang et al. [92], who manufactured Bi_2_O_3_/CeO_2_ Z-scheme heterojunctions in TiO_2_ nanotubes (NTs) by the hydrothermal deposition method and analyzed their effectiveness in degrading the dyes rhodamine B (RhB) and methylene blue (MB). After applying a voltage of 1.0 V to the target solution and 180 min exposure to a Xe lamp with a 1.5 AM filter, the results revealed that the compound TiO_2_ NTs/Bi_2_O_3_/CeO_2_ in the ratio (2:1) achieved the best result in removal of RhB and MB, with efficiency of 91.86% and 86.77%, respectively.

**Figure 8:**
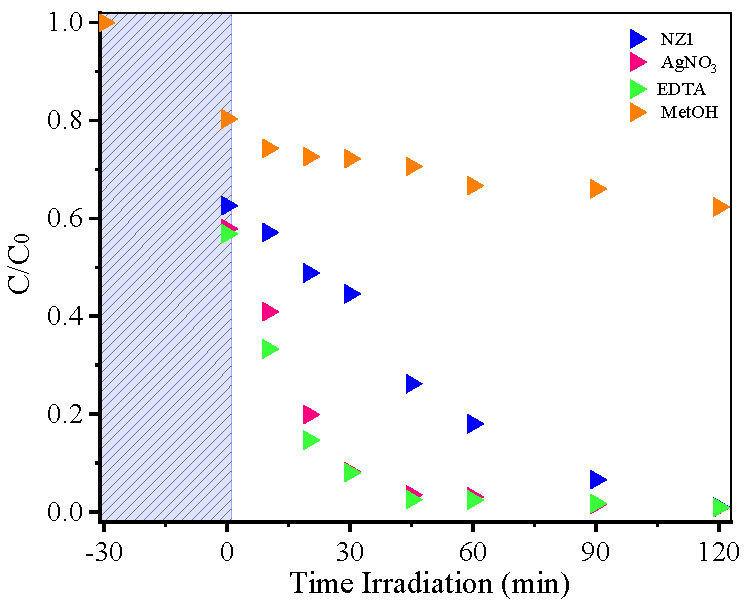
Evolution of MB photocatalysis by NZ1 sample in inhibitor test.

To study the mechanisms involved in MB photodegradation over the NZ1 sample, we used AgNO_3_, EDTA, and methyl alcohol as inhibitors of electrons, holes, and hydroxyl radicals, respectively (Figure 8). Comparing the results obtained from each inhibitor agent, we observed that MB removal became more difficult when MetOH was used as the inhibitor. This result suggests that the hydroxyl radicals play a vital role in MB degradation mediated by the NZ1 photocatalyst. On the other hand, in the presence of EDTA and AgNO_3_, the dye degradation remained unchanged. The degradation rate was 22.4% using MetOH and ~98.4% using EDTA or AgNO_3_.

Based on previously published studies, we propose a mechanism for MB degradation by NZ1 (Equations (7)–(13)). As clarified in the literature, the photoactivation of a semiconductor with adequate radiation promotes the jump of the electron from the valence band (VB) to the conduction band (CB), thus forming electron/hole pairs [93]. Once these charge carriers are generated, they may recombine or migrate to the surface, where redox reactions occur and will cause degradation of the target pollutant. For example, holes in the valence band react with adsorbed water molecules, while electrons present in the conduction band react with adsorbed molecular oxygen on the surface of the material. Different oxidant species, such as superoxide and hydroxyl radicals, have high reactivity and low selectivity. As a result, these species cause the breakdown of bonds in organic compounds and the subsequent degradation of pollutants.

*ZnNiO + hν* → *h^+^_(VB)_ + e^−^_(CB)_* (7)

*h^+^_(VB)_ + H*_2_*O _(adsorbed)_* → *H^+^ + ^•^OH*
*_(adsorbed)_* (8)

*e^−^_(CB)_ + O*_2_ → *^•^*
*O*_2_
*^−^* (9)

*^•^O*_2_*^−^  + H*_2_*O*_2_ → *^•^OH + OH^−^ + O*_2_ (10)

*Ni*^2*+*^*+ e^−^* → *Ni^+^* (11)

*Ni^+^ + O*_2_ → *Ni*
^2*+*^
*+ ^•^O*_2_
*^−^* (12)

*MB + ^•^OH* → *degradation/discoloration* (13)

**Figure 9:**
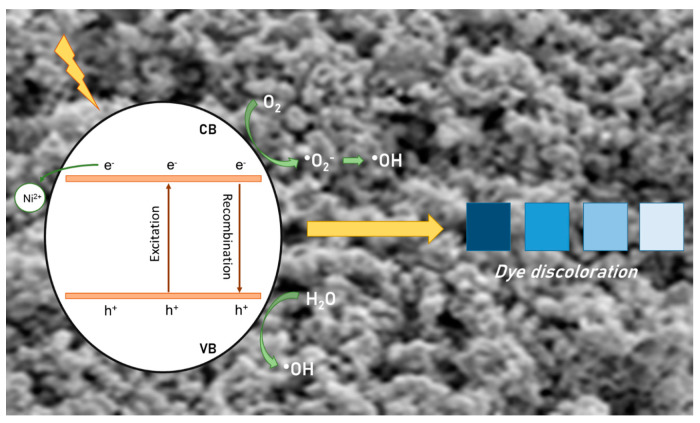
Proposal mechanism of MB degradation/discoloration by NZ1 sample under UV light.

The photocatalytic activity of a semiconductor is directly influenced by the concentration of charges on its surface as well as surface defects, which can affect the charge recombination frequency [12,38,41]. The introduction of Ni cations dopant can create structural defects that act as intermediates for electron energy levels and electron acceptors. This, in turn, delays the recombination process of electron/hole pairs. The XRD, DRS, Raman, and PL results demonstrate the formation of these defects, which are crucial for the semiconductor’s photocatalytic performance. The possible mechanism of action of the semiconductor can also be represented by the scheme in Figure 9.

**Figure 10:**
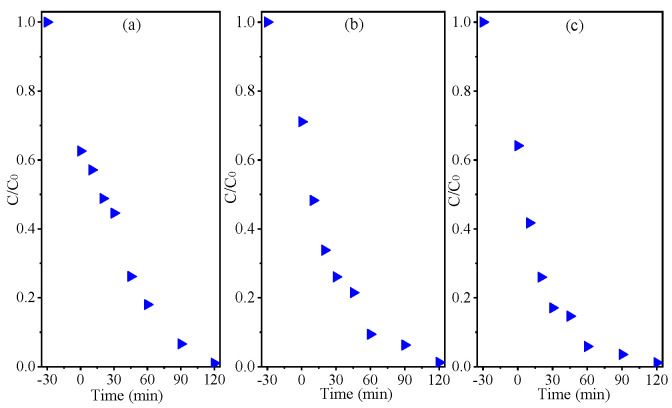
Reuse test for NZ1 photocatalyst: (**a**) first; (**b**) second; and (**c**) third cycles.

On the other hand, the stability of photocatalysts can be assessed by conducting reuse tests. In this study, the recovery and reuse of the material were evaluated by repeating the photocatalytic experiments three times, and the results were plotted in Figure 10a–c, which shows the concentration variation versus irradiation time. After three cycles, it was observed that the Ni-doped ZnO material (NZ1) was able to maintain its ability to remove MB, with a degradation rate of approximately 98.2% after the second and third reuse.

**Figure 11:**
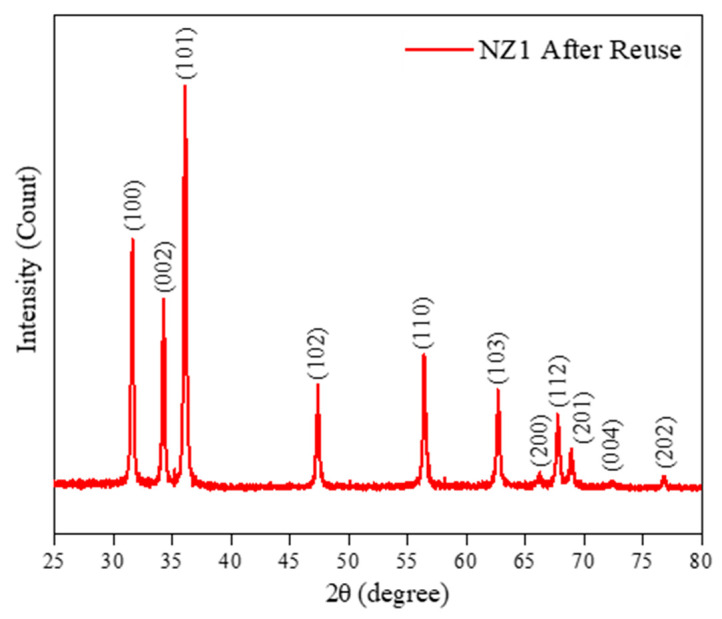
Powder diffractogram of NZ1 sample after the third cycle of reuse.

This indicates that the NZ1 material has high stability and can be reused multiple times, reducing photocatalyst production costs. These findings are significant for producing cost-effective photocatalysts. Finally, the recyclability of the material is linked to its structural stability. To check whether structural changes had occurred in the NZ1 material after the third reuse cycle, the dust collected was analyzed using the XRD technique (Figure 11). The results indicate that the main peaks of the material’s crystalline structure remain unchanged. The diffraction peaks which correspond to the planes’ reflection (100), (002), (101), (102), (110), (103), (200), (112), (201), (004), and (202) are indexed to the wurtzite hexagonal structure of ZnO, according to the JCPDS data (Card No. 36-1451). Stability tests suggest that the recovery of the photocatalyst and its subsequent use do not affect the sample’s structural lattice [35,94,95].

## 3. Materials and Methods

### 3.1. Reagents and Cashew Gum Obtention

The raw materials used for the synthesis of photocatalyst nanoparticles (NPs) were zinc nitrate hexahydrate (Aldrich-Sao Paulo, Brazil, 99.0%), nickel nitrate (Aldrich- Brazil, 99.0%), ammonium hydroxide (Aldrich, 99.0%), and distilled water. For the photocatalytic tests, methylene blue (Dinamic-Barra da Tijuca, Brazil, 97.0%), methyl alcohol (Neon-Sao Paulo, Brazil, 99.5%), ethylenediamine tetraacetic acid (EDTA) (Dinamic- Brazil, 99.0%), and silver nitrate (Vetec-Duque de Caxias, Brazil, 99.0%) were employed. 

For the cashew gum obtention, the nodules were collected from cashew trees (*Anacardium occidentale* L.). Isolation and purification were carried out as suggested in the literature. After removing impurities, the material was dissolved in distilled water (1:10 *m*/*v*) under mechanical agitation for 24 h. Then, the pH of the solution was adjusted (pH = 7) with the addition of NaOH solution. The gum was precipitated in ethanol, centrifuged, and washed with acetone three times. Finally, the gum obtained was dried in an oven at 50 °C for 24 h.

### 3.2. Green Synthesis of Zn_1−x_Ni_x_O Compound

**Figure 12:**
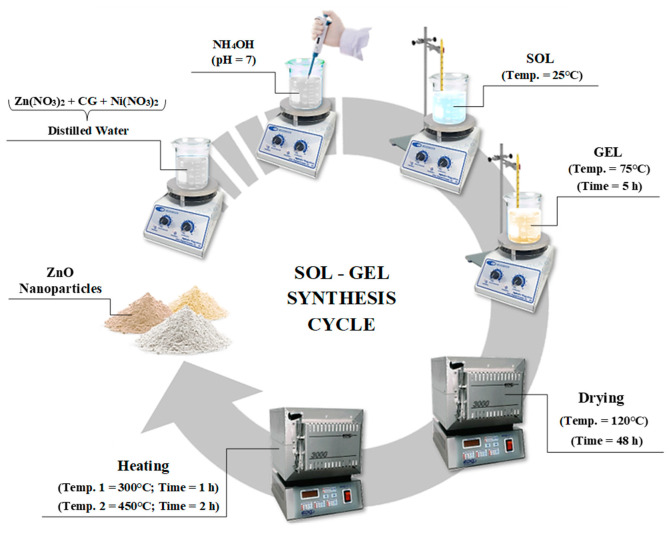
Illustrative scheme of the synthesis process of Zn_1−x_Ni_x_O compound.

The synthesis of the nanoparticles with composition Zn_1-x_Ni_x_O (x = 0.00, 0.01 and 0.03) and named as NZ0 (for pure ZnO), NZ1 (for Zn_0.99_Ni_0.01_O) and NZ3 (for Zn_0.97_Ni_0.03_O), was carried out using the sol–gel method. The illustrative scheme of the synthesis process is shown in Figure 12.

Initially, 1.0 g of the natural polysaccharide cashew gum (CG) and precise quantities of the Zn(NO_3_)_2_ and Ni(NO_3_)_2_ reagents were weighed to obtain a solution with a concentration of 0.7 mol·L^−1^ in 100 mL of distilled water, which were kept under mechanical stirring for 1 h. After adjusting the pH to 7 with NH_4_OH, the solutions were heated to 75 °C for 5 h under constant stirring to form the gel. Finally, the resulting products were dried for 48 h at 120 °C and then calcined: first, for 1 h after reaching a temperature of 300 °C and then, for 2 h after reaching 450 °C. The entire calcination process was carried out with a heating ramp of 1 °C min^−1^. Doped and undoped samples were obtained under the same synthesis conditions. The final products were named according to the dopant percentage: NZ0 (for ZnO); NZ1 (for the sample doped with 1% Ni); and NZ3 (for the sample doped with 3% Ni).

### 3.3. Characterization Technique

The structural characterization of the compounds was carried out using an X-ray diffractometer, model D8 Advance from Bruker (Billerica, MA, USA) with Cu-Kα radiation (λ = 1.5406 Å), and monochromator and configured in a Bragg-Brentano type geometry in a $25^{{}^{\circ}}\leq 2\theta \leq 80^{{}^{\circ}}$ range and 0.02 step size. The Raman measurements were obtained in a Raman spectrometer, model Santerra from Brunker, and excited using a solid-state laser through the 532 nm line. Diffuse reflectance spectra were measured using a UV-VIS spectrophotometer, Shimadzu (Columbia, MD, USA), UV-2700 using integrating spheres with 150 mm of diameter, while photoluminescence (PL) measurements were obtained in a Spectrofluorometer Horiba—Jobin Yvon Fluorolog-3 and at an excitation wavelength of 340 nm with a High-Resolution Spectrometer—HR4000CG-UV-NIR—Ocean Optics. Finally, the micrographic of the samples was obtained via a scanning electron microscopy (SEM) model TESCAN MIRA3 with an energy-dispersive X-ray spectrometer (EDS) coupled.

### 3.4. Photocatalytic Tests

For Ni-doped ZnO samples, the photocatalytic tests were performed using an aqueous solution of the dye methylene blue (MB) as a model contaminant, whose concentration was 1.0 × 10^−5^ mol·L^−1^. For each experiment, a concentration of 0.5 g·L^−1^ of the photocatalyst compound was added to 100 mL of the MB dye solution in a borosilicate reactor and exposed to UV radiation from a commercial lamp of 160 W, with a radiation intensity of 800 Lux. All tests were carried out under magnetic stirring and temperature control of 25.0 ± 1.0 °C. After maintaining the system in the dark for 30 min to attain adsorption/desorption equilibrium, it was subjected to radiation for 120 min. During the irradiated reaction, small amounts were extracted at regular intervals and separated by centrifugation, using three cycles at 5000 rpm for 2 min each. MB dye concentration was monitored using the absorption band at 664 nm, and its percentage degradation was calculated via Equation (14).

(14) $$Degradation\;\left(\%\right)=\frac{C_{0}-C}{C_{0}}\times 100$$

Here, *C*_0_ and *C* correspond to the initial and final concentration of the dye, respectively. In addition, the reusability of the semiconductor was evaluated by three consecutive cycles under the same conditions as the photocatalytic tests, where 0.5 g L^−1^ of catalyst and 100 mL of the dye solution were used with 1.0 × 10^−5^ mol·L^−1^of the concentration. For each experiment, a new pollutant solution was used, and the catalyst was separated, washed, and dried for subsequent reuse. To investigate the role of different reactive species in the photocatalytic process, studies of radical scavengers were carried out. In this sense, Ethylenediaminetetraacetic acid (EDTA) (2.4 × 10^−6^ mol·L^−1^), methyl alcohol (3.4 × 10^−6^ mol·L^−1^), and AgNO_3_ (5.0 × 10^−4^ mol·L^−1^). In this case, the tests were also carried out under the same conditions as the photocatalytic tests, only adding specific amounts of inhibitors at the beginning of each reaction.

## 4. Conclusions

The sol–gel method and cashew gum polysaccharide were used to synthesize pure and Ni-doped ZnO nanoparticles successfully. XRD and Raman analyses established that ZnO had a typical hexagonal wurtzite structure, and no secondary phases were detected. Distortions were observed in the ZnO crystal lattice, which are associated with the difference between the ionic radii of the Ni and Zn cations and which are reflected in changes in the crystallite size (61.9–81.5 nm). These distortions increased the density of vacancy defects in the structure of the compounds, which affected the optical and photoluminescence properties, as indicated by DRS and PL analyses. SEM analyses revealed that the systems are modeled by clusters of spherical particles. In the photocatalytic tests, the NZ1 sample showed better performance in removing the MB dye (98.4%), indicating that the ideal dopant concentration can influence the photocatalytic performance. MB removal was attributed to •OH radicals based on inhibitor tests. This compound also remained stable during free reuse tests, maintaining a photocatalytic efficiency of 98.2%. Therefore, within the established conditions, the doped materials had excellent efficiency in MB degradation and showed promise for new photocatalytic studies. Furthermore, the successful use of cashew tree gum in the NP synthesis process stands out, proving to be a very viable and innovative alternative, mainly due to its low cost and reduced environmental impact, which can be explored in new works for various syntheses of other materials.

## Figures and Tables

**Table 1:** Structural parameters obtained from the XRD patterns.

Samples	Lattice Parameters (Ǻ)		*c/a*	Crystallite Size *D* (nm)	Bond Length *L* (Ǻ)	Dislocation Density δ × 10^−3^ (nm^−2^)	Lattice Strain ε (%)
	*a* = *b*	*c*					
NZ0	3.2409	1.6023	61.90	1.9725	1.9725	0.261	0.036
NZ1	3.2379	1.6024	61.62	1.9706	1.9706	0.263	0.145
NZ3	3.2482	1.6011	81.56	1.9764	1.9764	0.150	0.258

**Table 2:** Frequencies assigned to possible phonon modes for the Zn_1−x_Ni_x_O system.

Assignments	Wavenumber (cm^−1^)		
	NZ0	NZ1	NZ3
E_2_^low^	99	99	99
2E_2_^low^	214	205	202
B_1_^low^	271	272	273
E_2_^high^ − E_2_^low^	331	329	327
A_1_(TO)	377		
E_1_(TO)	411		
E_2_^high^	438	436	435
B_1_^high^	536	535	535
A_1_(LO)	581	580	578
E_1_(LO)	660	659	657

**Table 3:** Comparison between the reaction rate constant, *k*, obtained for NZ1, NZ3, and some materials discussed in other texts using the MB pollutant.

Photocatalysts	MB Initial Concentration	Catalyst Dosage	Light Source	k (min^−1^)	References
NZ1 NPs	1.0 × 10^−5^ mol·L^−1^	0.5 g·L^−1^	UV (160 W)	2.95 × 10^−2^	Present Work
ZnO Nanowalls	1.0 × 10^−5^ mol·L^−1^	50 mM	UV (10 W)	1.63 × 10^−2^	Ref. [82]
Nd:ZnO (0.1%)	6 mg·L^−1^	2 g·L^−1^	Vis (400 W)	4.44 × 10^−3^	Ref. [83]
ZnO − NPs	10 mg·L^−1^	0.5 g·L^−1^	Sunlight	4.78 × 10^−2^	Ref. [84]

## Data Availability

Data are contained within the article.
